# Acting as Change Agents: Insight Into Québec Occupational Therapists’ Current Practice: Actions menées à titre d’agents de changement : aperçu des pratiques actuelles parmi les ergothérapeutes du Québec

**DOI:** 10.1177/0008417421994367

**Published:** 2021-03-08

**Authors:** Annie Carrier, Alexandra Éthier, Michaël Beaudoin, Anne Hudon, Denis Bédard, Emmanuelle Jasmin, France Verville

**Keywords:** Advocacy, Change agency, Professional practice, Agent de changement, défense des intérêts, pratique professionnelle

## Abstract

**Background.:**

Change agents’ actions have been studied mainly from a theoretical perspective.

**Purpose.:**

This study aimed to empirically identify occupational therapists’ actual change agent actions.

**Method.:**

As part of a research partnership with the Canadian Association of Occupational Therapists-Québec chapter, we conducted this cross-sectional pilot study using an online survey.

**Findings.:**

The change agent practices of our 103 participants involve many types of actions but show underinvestment in mass communication. Mass communication actions are more frequent when participants have greater experience, additional academic degrees, and training in change agency. Also, occupational therapists with additional academic degrees and change agency training tend to use a wider variety of actions. Finally, our participants’ actions principally target actors in the clinical context, rarely political actors.

**Implications.:**

Our results suggest that occupational therapists can and will invest in the full range of change agent actions provided they can acquire the necessary knowledge and skills.

## Abstract (fr)

**Contexte de recherche.:**

Les actions d’agent de changement (AC) ont surtout été étudiées sous un angle théorique.

**Objectif de la recherche.:**

L’objectif de cette recherche était de déterminer les actions d’AC posées par les ergothérapeutes.

**Méthodologie.:**

Dans le cadre d’un partenariat de recherche avec le chapitre québécois de l’Association canadienne des ergothérapeutes, nous avons mené cette étude-pilote transversale à l'aide d’un sondage en ligne.

**Résultats.:**

La pratique d’AC des 103 participants implique plusieurs types d’actions, mais la communication de masse est sous-investie. Cependant, un niveau d’expérience plus grand, des diplômes supplémentaires et une formation d’AC favorisent son déploiement. De plus, les ergothérapeutes avec diplômes supplémentaires ou formation d’AC varient davantage les actions utilisées. Enfin, les actions des participants s’adressent principalement aux acteurs du contexte clinique et rarement aux acteurs politiques.

**Implications.:**

Nos résultats semblent suggérer que les ergothérapeutes peuvent investir l’entièreté des actions d’AC et le feront pour autant qu’ils puissent développer les connaissances et les compétences requises.

## Introduction

Occupational needs that are met support good health and well-being, while occupational injustice can lead to health inequities (Hammell & Iwama, 2012). Periods of uncertainty have the potential to increase these injustices (Smith & Judd, 2020). The COVID-19 pandemic is a prime example of this: it revealed or exacerbated health and healthcare inequities that already existed in populations at risk of increased vulnerability, including elderly adults and people with disabilities, chronic conditions, or mental health problems (Smith & Judd, 2020). Specifically, the pandemic aggravated in Québec the long-standing occupational deprivation experienced by seniors in long-term care, which was publicly revealed by 146 occupational therapists (Lacoursière, 2020). These occupational therapists called for systemic changes to better support meaningful occupations. To bring about systemic changes, since occupations are social and political in nature, Laliberté Rudman (2014; 2020) suggests that occupational therapists embrace a transformative approach, including a critical stance, and act as change agents.

The Canadian Association of Occupational Therapists (CAOT) Profile of Practice (2012) frames occupational therapy practice in Canada. It defines change agents as occupational therapists who “use their expertise and influence responsibly to advance occupation, occupational performance, and occupational engagement” (CAOT, 2012, p. 3). This definition can be criticized for its focus on performance (see for example Hammell, 2017), its lack of consideration of occupational well-being and needs (Doble & Santa Caron, 2008), and its lack of details regarding the dimensions of the change agent role (Carrier & Beaudoin, 2020). With the specific aim of exploring these dimensions, in a scoping study of the literature, Carrier and Beaudoin (2020) defined the change agent role as a continuum between two main configurations: clinical and social. For each configuration, the people involved, the objectives sought, the actions carried out, and the context in which these actions occur vary.

Acting on a microsystemic level, clinical change agents mainly aim to respect and defend the values and choices of an individual patient or group, including when organizational policies or healthcare team objectives threaten these values and choices (Carrier & Beaudoin, 2020). Social change agents, on the other hand, expand their actions to the macrosystem, with the objective of influencing health and healthcare-related norms and regulations to promote occupational justice for a community or population (Carrier & Beaudoin, 2020). To meet their objectives, change agents use a wide range of actions that include: providing information about rights and options; discussing with and making demands of other professionals or managers; advocating in an organized association (e.g.: patients’ rights or professional associations, political groups, unions, etc.); using media; convincing politicians; and advocating with vulnerable or marginalized populations and on their behalf (Carrier & Beaudoin, 2020). The broad spectrum of change agent actions could even go so far as to include civil disobedience (Bennett et al., 2020).

Although interesting, the results from Carrier and Beaudoin’s scoping study (2020) do not provide information on the actual change agent actions of occupational therapists in their everyday practice. In their scoping study, more than two thirds (69.7%) of the articles selected were not empirical studies (opinion piece, reflection based on grey literature, unsystematic literature review, etc.). Furthermore, although previous empirical studies explored occupational therapists’ reasons for advocacy actions (Dhillon et al., 2010) and experiences of advocacy activities (Dhillon et al., 2016), to our knowledge, no empirical study has investigated the clinical practice of occupational therapists to identify their concrete change agent actions. Since acting as change agents is considered uncomfortable for most occupational therapists, whether experienced (Finlayson, 2013; Restall & Ripat, 2008) or novice (Rochette et al., 2020), knowing more about what actually happens in practice is of particular interest. More specifically, results might inform educators and professional stakeholders regarding what the objective of future training efforts should be. In this pilot study, therefore, we aimed to identify occupational therapists’ actual change agent actions.

## Method

We conducted this pilot study as part of a research partnership with the Canadian Association of Occupational Therapists-Québec chapter (CAOT-Qc); the study took place from September 2018 to October 2019. In this initiative, we provided Québec occupational therapists with a one-day training session on the change agent role in occupational therapy and asked them to evaluate this training. This paper presents sociodemographic information and actual change agent actions used by occupational therapists who participated in the training; it does not discuss the training session assessment. For details on the training session and its evaluation, see Rahimaly and colleagues (2019).

### Sampling and Recruitment

After obtaining ethics approval from the CIUSSS de l’Estrie–CHUS ethics committee (2019-2938 obtained July 23, 2018), participants were recruited via emails from CAOT-Qc and the Ordre des ergothérapeutes du Québec. In accordance with Dillman et al.’s (2014) recommendations, we emailed potential participants three times at different times on different days. We also posted a recruitment flyer on the Facebook pages of Ergothérapie Québec (approx. 5,000 members) and CAOT-Qc (approx. 500 members in 2018). Initially, 217 occupational therapists emailed the research team to indicate their interest in participating in the one-day session. All occupational therapists had an entry-level diploma in occupational therapy. In Québec, that means that occupational therapists either had a bachelor’s (if they graduated before 2010) or a bachelor’s and master’s degree in occupational therapy after 2010. On a first-come, first-served basis, a research assistant (RA) contacted potential participants by phone to explain the research project and answer their questions. If they agreed to participate, the RA collected the necessary information to email them the consent form and gave them a choice of location for the training session. In all, 119 participants confirmed their attendance at the different training sessions. The others did not participate for various reasons (distance, date, group was full, etc.). Of the 119 confirmed participants, 16 opted out at the last minute (illness, accident, family obligation, etc.), reducing the total to 103 participants. As this training was part of a research project, we obtained written consent from all the participants via email or in person at the beginning of the training day.

### Data Collection

In the training session, the Change Agent Actions Planning model (CAAP model; Carrier & Contandriopoulos, 2016; Rahimaly et al., 2019) was presented to participants. The CAAP model comprises an eight-step systematic process for analyzing and planning change agent actions. Before ending the session, we asked participants to complete an online survey. This survey comprised 37 closed and open-ended questions, divided into three sections: (a) sociodemographic information (n = six questions with multiple-choice or open-ended answers regarding gender, age, years of experience as occupational therapists, additional academic degrees, additional training that participants felt was linked to change agency, and practice setting); (b) change agent practices (n = 2 multiple-choice questions with the option of adding a detailed answer regarding change agent actions); and (c) assessment of the training received (n = 29 questions). In the sociodemographic section, open-ended questions were as follows: “Do you have an additional diploma? If yes, specify” and “What additional training have you taken regarding the change agent role?” In the change agent practices section, multiple-choice questions were as follows: “Have you ever acted as a change agent in your profession with [multiple choices of actors]” and “please check the actions you have taken in situations where you acted as a change agent”. The open-ended question was “Please specify for the “other” category”. Multiple answers were permitted.

To determine relevant sociodemographic characteristics and change agent actions to include in the questionnaire, we relied on the scientific literature. For example, the clinical reasoning literature supported the inclusion of questions regarding training and years of experience. Since Carrier and Beaudoin’s conceptualization was not yet published, we mainly relied on the CAAP model, advocacy literature, and the CAOT (2012) Profile of Practice to determine examples of change agent actions to include in the questionnaire. The final selection was approved by all team members. To assess the training, we adapted the Participant Workshop Evaluation Questionnaire validated by Dagenais and colleagues (2012). Two methodological and content experts validated the content of our survey. Specifically, we informed the experts in writing of the study rationale, objectives, and method, and asked them to evaluate each question and the choice of answers for clarity, consistency, and comprehensiveness. They could also suggest alternative formulations. No iteration was necessary.

To host our survey, we used the institutional LimeSurvey platform. Eighteen of the participants had to complete a hard copy of the survey due to problems with their computers. An RA subsequently transcribed their answers into the online form. In this brief report, we present results from the participants’ answers to the sociodemographic and change agent practices sections only.

### Data Analysis

First, for the multiple-choice questions on participants’ sociodemographic characteristics and current practices, we used descriptive statistics (frequencies and percentages for categorical variables, and means and standard deviations for continuous variables). Second, using content analysis (Mays et al., 2005), we also categorized two of the three open-ended questions inductively using Microsoft Excel. Two team members (AE and MB) first read the entire set of answers, then coded the data. For example, data for additional diplomas led to codes that included degree-level and broad discipline (e.g., bachelor of social science and bachelor of education). We aggregated all similar codes into categories (Mays et al., 2005). For additional diplomas, they were aggregated according to degree level. We then assigned a number to each category, which was tagged beside each corresponding code in the Excel document. This enabled us to count frequencies. The third open-ended question (other change agent actions) was not coded since participants reported only seven “other” actions. For statistical analysis purposes, we kept the category “other” as is. Finally, through discussion and consensus, three members of the team (AC, AE, and MB) determined if each action and targeted actor related to the clinical, the social or both configurations of the change agent role.

Since our sample was small, we ran Fisher’s exact test (McDonald, 2014; Siegel, 1956). In our analysis, Fisher’s exact test was used to determine if years of practice, additional academic degrees, or additional training in change agency were associated with the type of change agent actions taken or type of actors targeted by these actions. Finally, we also ran variance analyses to see if the number of actions taken as a change agent or the number of actors targeted by the actions differed according to years of practice, additional academic degrees, or additional training in change agency. All results were considered significant when *p* was less than 0.05. We used SPSS 26 for the statistical analysis.

## Findings

In all, 103 occupational therapists participated in the training. Most participants were female (*n* = 95, 92.2%; Table 1) and at least 35 years old (*n* = 57, 55.3%). Of the 98 participants who reported their years of practice, many were experienced, meaning that they had practised as occupational therapists for at least 11 years (*n* = 58, 59.2%). Participants worked or had worked in 2.78 practice settings on average (*SD* = 1.6; data not shown). The most frequent settings were rehabilitation, community care, and hospital internal settings (Table 1). More than half of the 103 participants had an entry-level diploma in occupational therapy only (*n* = 61, 59.2%). Of the 42 participants with at least one academic degree other than their occupational therapy diploma (data not shown), the higher academic degrees obtained were distributed as follows: bachelor’s (*n* = 10, 9.7%), master’s (*n* = 26, 25.2%), PhD (*n* = 4, 3.9%), and not specified (*n* = 2; 1.9%). These 42 participants had a total of 54 diplomas in various disciplines (data not shown), that is, health (*n* = 17, 31.5%), biology (*n* = 11, 20.4%), social sciences (*n* = 10, 18.5%), education (*n* = 6, 11.1%), not specified (*n* = 5, 9.3%), management (*n* = 3, 5.6%), and engineering (*n* = 1, 1.9%). One third of participants (*n* = 34, 33.0%) had taken additional training related to the change agent role. This training involved occupational therapy interventions (e.g., workshop with CAOT), health and social services (e.g., specialized approach to senior care), communication (e.g., effective negotiation), management and administration (e.g., Lean method), health and social services policies, knowledge translation, and other (e.g., supervising interns).

**Table 1:** Participants’ Sociodemographic Characteristics by Years of Experience

	Total	Up to 5 Years	6–10 Years	11–20 Years	21-plus Years
	(*n* = 103)	(*n* = 24; 24.5%)Δ	(*n* = 16; 16.3%)Δ	(*n* = 33; 33.7%)Δ	(*n* = 25; 25.5%)Δ
	*n* (%)	*n* (%)Δ	*n* (%)Δ	*n* (%)Δ	*n* (%)Δ
**Gender**					
Female	95 (92.2)^1^	22 (22.4)	16 (16.3)	29 (29.6)	23 (23.5)
Male	7 (6.8)	2 (2.0)	0 (0.0)	3 (3.1)	2 (2.0)
Other	1 (1.0)	0 (0.0)	0 (0.0)	1 (1.0)	0 (0.0)
**Age (in years)**					
18–24	3 (2.9)^δ,1^	2 (2.0)	0 (0.0)	0 (0.0)	1 (1.0)^δ^
25–34	43 (41.7)^1^	17 (17.3)	9 (9.2)	13 (13.3)	3 (3.1)^δ^
35–44	28 (27.2)^1^	3 (3.1)	6 (6.1)	16 (16.3)	2 (2.0)
45–54	23 (22.3)^1^	2 (2.0)	0 (0.0)	4 (4.1)	16 (16.3)
55 or more	6 (5.8)^1^	0 (0.0)	1 (1.0)	0 (0.0)	3 (3.1)
**Additional degrees (y)**					
Bachelor’s	10 (9.7)^1^	2 (2.0)	1 (1.0)	4 (4.1)	2 (2.0)
Master’s	26 (25.2)	1 (1.0)	4 (4.1)	10 (10.2)	11 (11.2)
PhD	4 (3.9)^1^	1 (1.0)	0 (0.0)	2 (2.0)	1 (1.0)
Not specified	2 (1.9)^1^	1 (1.0)	0 (0.0)	0 (0.0)	0 (0.0)
**Additional training (y)**	34 (33.0)^1^	3 (3.1)	6 (6.1)	13 (13.3)	10 (10.2)
**Practice settings (y)**					
Rehabilitation	50 (48.5)^1^	8 (8.2)	7 (7.1)	17 (17.3)	15 (15.3)
Community care	47 (45.6)^1^	10 (10.2)	7 (7.1)	13 (13.3)	13 (13.3)
Hospital internal setting	43 (41.7)^1^	8 (8.2)	6 (6.1)	13 (13.3)	14 (14.3)
Academic setting	36 (35.0)	4 (4.1)	8 (8.2)	12 (12.2)	12 (12.2)
Other	38 (36.9)^1^	7 (7.1)	9 (9.2)	11 (11.2)	7 (7.1)
Long-term care	35 (34.0)^1^	7 (7.1)	2 (2.0)	7 (7.1)	17 (17.3)
Hospital external setting	33 (32.0)^1^	7 (7.1)	5 (5.1)	10 (10.2)	10 (10.2)
Youth education and protection	5 (4.9)^1^	1 (1.0)	2 (2.0)	1 (1.0)	0 (0.0)

ΔFor each cell, percentages were calculated based on 98 respondents (5 participants had missing data); ^d^One participant indicated being aged 18–24 years and having 21 years or more of experience, and two participants indicated being aged 25–34 years and having 21 years or more of experience; y = yes; ^1^This total includes participants who provided their years of experience (sum of the cells in this row) and participants who did not provide their years of experience (Δmissing data).

As change agents, participants engaged in many actions. The three most frequently reported clinical actions were communicating (*n* = 93, 90.3%), informing (*n* = 91, 88.3%), and collaborating (*n* = 87, 84.5%; Table 2). Participants rarely engaged in social actions related to mass communication channels such as writing opinion pieces, giving a speech, or talking to the media (Table 2). Although few reported using actions other than the choices proposed (*n* = 7, 6.8%; data not shown), when they did those actions were mainly social. They included teaching or giving presentations in the academic setting (*n* = 2, 1.9%), initiating a petition to the National Assembly (provincial legislature), designing tools for occupational therapists, creating an awareness campaign, participating in the reorganization of health and social services departments, and demonstrating or showing an example (respectively *n* = 1, 1.0%; data not shown). Actions used to communicate (*p* = .01), collaborate (*p* = .01), and give a speech (*p* = .01) differed according to years of experience. From the 98 participants who reported their years of experience, participants with between 11 years and 20 years of experience were those less likely to communicate (*n* = 8, 8.2%; data not shown) while participants with more than 21 years of experience were those who tended to have given a speech in their change agent role (*n* = 9, 9.2%).

**Table 2:** Participants’ Change agent Practices by Years of Experience and Role Configurations

	Total	Up to 5 Years	6-10 Years	11–20 Years	21-plus Years	*p-value*
	(*n* = 103)	(*n* = 24; 24.5%)Δ	(*n* = 16; 16.3%)Δ	(*n* = 33; 33.7%)Δ	(*n* = 25; 25.5%)Δ	
Categorical variable	*n* (%)	*n* (%)Δ	*n* (%)Δ	*n* (%)Δ	*n* (%)Δ	
**Actions taken by change agents (y)**						
Clinical						
Communicate	93 (90.3)^1^	22 (22.4)	16 (16.3)	25 (25.5)	25 (25.5)	.01*
Inform	91 (88.3)^1^	19 (19.4)	15 (15.3)	27 (27.6)	25 (25.5)	.08
Collaborate	87 (84.5)^1^	18 (18.4)	16 (16.3)	24 (24.5)	24 (24.5)	.01*
Social						
Create networks	41 (39.8)^1^	7 (7.1)	5 (5.1)	16 (16.3)	12 (12.2)	.36
Plan an event	37 (35.9)^1^	5 (5.1)	6 (6.1)	12 (12.2)	13 (13.3)	.17
Develop a program	32 (31.1)	5 (5.1)	7 (7.1)	13 (13.3)	7 (7.1)	.35
Write an opinion piece	20 (19.4)	5 (5.1)	3 (3.1)	6 (6.1)	6 (6.1)	.98
Give a speech	17 (16.5)	2 (2.0)	0 (0.0)	6 (6.1)	9 (9.2)	.01*
Talk to the media	9 (8.7)	2 (2.0)	2 (2.0)	2 (2.0)	3 (3.1)	.86
Clinical and social						
Convince	71 (68.9)^1^	19 (19.4)	10 (10.2)	20 (20.4)	18 (18.4)	.46
Argue	60 (58.3)^1^	15 (15.3)	6 (6.1)	18 (18.4)	18 (18.4)	.17
Create partnerships	58 (56.3)^1^	14 (14.3)	9 (9.2)	15 (15.3)	16 (16.3)	.54
Plan actions as change agent	50 (48.5)^1^	12 (12.2)	7 (7.1)	13 (13.3)	15 (15.3)	.47
Advocate	49 (47.6)^1^	13 (13.3)	7 (7.1)	12 (12.2)	12 (12.2)	.60
**Actors targeted by actions (y)**						
Clinical						
Patients’ families	84 (81.6)^1^	18 (18.4)	14 (14.3)	25 (25.5)	23 (23.5)	.31
Colleagues	76 (73.8)^1^	18 (18.4)	15 (15.3)	20 (20.4)	21 (21.4)	.06
Low-level or middle managers	64 (62.1)^1^	12 (12.2)	10 (10.2)	22 (22.4)	18 (18.4)	.45
Social						
Senior managers	56 (54.3)^1^	12 (12.2)	5 (5.1)	18 (18.4)	17 (17.3)	.15
Low-level political actors	15 (14.6)	3 (3.1)	2 (2.0)	6 (6.1)	4 (4.1)	.98
High-level political actors	8 (7.8)	2 (2.0)	1 (1.0)	3 (3.1)	2 (2.0)	.99
Never took any actions	3 (2.9)	1 (1.0)	0 (0.0)	2 (2.0)	0 (0.0)	.70
Continuous variables	*M (SD)*	*M (SD)* **Δ**	*M (SD)* **Δ**	*M (SD)* **Δ**	*M (SD)* **Δ**	*p value*
**Number of actions taken as change agents**	7.00 (3.17)	6.67 (3.16)	6.81 (2.17)	6.36 (3.66)	8.24 (3.07)	.15
**Number of actors targeted by change agent actions**	2.97 (1.21)	2.75 (1.11)	2.94 (0.68)	2.91 (1.44)	3.40 (1.26)	.28

**Δ**For each cell, percentages or means were calculated based on 98 respondents (5 participants had missing data); *p < 0.05; ^1^This total includes participants who provided their years of experience (sum of the cells in this row) and participants who did not provide their years of experience (Δmissing data).

Actions also varied according to whether participants had an additional academic degree or not (data not shown). When they did, social actions such as creating networks (*p* = .00) and giving a speech (*p* = .03) were more frequent. Finally, creating networks (*p* = .03), creating partnerships (*p* = .01), giving a speech (*p* = .02), and planning an event (*p* = .00) tended to happen more often when participants had taken additional training related to the change agent role (data not shown).

Most participants reported using more than one action as change agent, with an average of 7.00 actions (*SD* = 3.17; Table 2). The number of actions taken as change agent did not differ significantly according to years of experience (respectively *F* (3, 94) = 1.803, *p* = .15) but did differ when participants reported having another academic degree (*F* (1, 101) = 8.308, *p* = .01) or having received additional training related to the change agent role (*F* (1, 101) = 5.559, *p* = .02). Participants with an additional academic degree (*M* = 8.05, *SD* = 2.95) or training (*M* = 8.02, *SD* = 3.42) reported using more actions than those who did not have an additional academic degree (*M* = 6.28, *SD* = 2.95) or training (*M* = 6.49, *SD* = 2.93; data not shown).

Finally, the 103 participants mostly acted as change agents with patients’ families (*n* = 84, 81.6%), followed by their colleagues (*n* = 76, 73.8%) and their low-level or middle managers (*n* = 64, 62.1%; Table 2), all actors in the clinical configuration. They less frequently targeted social configuration actors like senior managers (*n* = 56, 54.3%), low-level political actors such as city counsellors, members of parliament, or political party representatives (*n* = 15, 14.6%), or high-level ones such as ministers or the prime minister (*n* = 8, 7.8%). Actors targeted by actions did not vary significantly according to years of experience, additional academic degrees or additional training (data not shown). On average, participants acted as change agents with 2.97 different types of actors (*SD* = 1.21). The number of actors targeted by change agent actions did not differ significantly according to years of experience (*F* (3, 94) = 1.304, *p* = .28), having another academic degree (*F* (1, 101) = 3.556, *p* = .06; data not shown) or additional training (*F* (1, 101) = 1.938, *p* = .17; data not shown).

## Discussion

We wanted to paint a picture of the current practice of occupational therapists in their role as change agents. Our results show that this practice involves many types of actions but most could be linked to Carrier and Beaudoin’s (2020) clinical configuration of the change agent role (e.g., communicate, inform, and collaborate). There is underinvestment in mass communications, such as giving speeches and talking to the media, which could be linked to the social configuration (Carrier & Beaudoin, 2020). However, these types of actions are used more with increasing experience, additional academic degrees, and change agency training. Also, occupational therapists with additional academic degrees and change agency training tend to use a wider variety of actions, including networking, creating partnerships, and planning events. In our participants’ case, change agent actions mainly target actors in the clinical context (patients’ families, colleagues, and managers), less so actors in the political arena, whether municipal, provincial, or federal. Our participants mostly enacted the clinical configuration of the change agent role. The survey design and results obtained do not allow us to determine why there was less embracing of the social configuration. Nevertheless, because of the importance of fully occupying the change agent role to increase occupational justice, it is useful to explore explanatory hypotheses. Various hypotheses could be explored including the following. Everyday occupational therapy practice occurs within the clinical context and that is where opportunities for change agency arise; there might be little time to invest in actions outside the clinical context; and experience and additional education and training might increase social prestige, power, and networking, which could generate more opportunities to act as social change agents. We will limit ourselves to two hypotheses addressed in the literature: one related to individuals, that is, knowledge and skills required to act as change agents outside the clinical context, and the other systemic in nature, that is, possible effects of New Public Management (NPM).

Not surprisingly, occupational therapists’ change agent practices include mainly communication and collaboration actions. According to Rochette and colleagues (2020), recent occupational therapist graduates in Québec are very comfortable with enacting communicator and collaborator competencies in the clinical setting. Carrier and Beaudoin (2020) noted that the clinical configuration of the change agent role is more aligned with the usual work of occupational therapists, whereas the social configuration involves different types of knowledge and skills. As there is usually less emphasis on teaching and mobilizing these types of “social” knowledge and skills during entry-level education (Bouvrette, 2017; Rahimaly et al., 2019), this might explain, at least in part, why our participants reported least investment in mass communication channels. However, since participants with greater experience or additional education indicated using these types of actions more often, our results suggest that occupational therapists can and will invest in the social configuration of their change agent role provided they can develop the requisite experience, knowledge, and skills.

Our results could also be considered in light of NPM and its underlying neoliberal values, currently operating in Québec (Carrier, 2020). Based on the premise that public healthcare services should be managed like private enterprises (Nordgren, 2008), NPM involves a top-down view of management and economic discourse (Carrier, 2020), and it supports the current vision of professionals’ duty of loyalty (Lampron, 2020). The effect of the duty of loyalty and an “omerta syndrome” (code of silence) in the healthcare system (Lampron, 2020) might explain why, in their change agency endeavors, participating occupational therapists rarely target actors outside the realm of clinical settings. Fear of negative repercussions could be rooted in part (see further) in the confidentiality agreement that new employees must sign, in disciplinary proceedings for professional misconduct (e.g., case of Carolyn Strom in Saskatchewan; Strom v Saskatchewan Registered Nurses’ Association, 2020), and in disciplinary action by the employer (e.g., Québec nurses suspended without pay by their organization; Bouchard, 2020). Although legislators are being called upon to amend the law to clarify the notion that professionals’ duty of loyalty should be to the public (Lampron, 2020), as things stand now, health professionals remain generally unprotected (Perron et al., 2020).

Documenting NPM’s effects on professional practice, Drolet and colleagues (2020) suggested that fear of negative repercussions leads professionals to submit to organizational standards they might disagree with, instead of acting to change them. Lack of power, as an individual or profession (Clark, 2010), might explain this fear. If occupational therapists feel powerless or disempowered in the face of senior managers or political structures, they are likely to act as change agents in the context where they feel they can exert influence, that is, the clinical setting. Furthermore, according to Drolet and colleagues (2020), NPM leads professionals to implicitly adopt neoliberal values and come to think about their “work in individual terms, where individual performance takes precedence over adequately addressing community needs” (free translation; Drolet et al., p. 104). Considering that many occupational and social injustices have systemic origins (Hammell & Iwana, 2012), individualistic change agency could lead to repeated but ultimately ineffective change agent actions. This reveals even more clearly the need for training based on a systems mind-set (Hubinette et al., 2016).

### Implications for Occupational Therapy Practice and Future Research

Our results show that if occupational therapists are to fully assume their role as change agents, one avenue could be to invest more resources in teaching social configuration knowledge and skills. For example, medicine (Hubinette et al., 2016), physical therapy (Bessette et al., 2020), and occupational therapy (Bouvrette, 2017) have all identified advocacy skills as an area that should be invested in. Whether this teaching should be included in the entry-level master’s degree, a professional PhD or continuing education courses is up for debate (Carrier & Beaudoin, 2020). Although Bouvrette has documented the teaching of advocacy skills in Québec and Ontario occupational therapy programs, further studies could cover all Canadian programs and document the full range of the change agent’s role, knowledge, and skills actually taught. Also, future research should document systematically and in more detail Canadian occupational therapists’ change agent actions in each configuration (clinical and social) and why they invest more in clinical change agent actions, paying particular attention to actions that may challenge the duty of loyalty. Education and current practice could then be compared to the conceptualization of the change agent role, with possible areas for improvement underlined in both practice and education.

### Strengths and Limitations

To our knowledge, this is the first study to empirically investigate occupational therapists’ change agent actions. Since we used a convenience sample, our results might not be representative of the practice of all occupational therapists in Québec. Also, because our sample was small, a type II error might have led to non-significant results. Another limitation was that the metrological properties of the questionnaire we used were not known. However, we did have its content validated by experts. Also, some questions were open-ended, which allowed for aspects not covered in the questionnaire to be specified (e.g., type of additional training, additional academic degrees, and other change agent actions). Filling out the questionnaires right after the training session resulted in an excellent completion rate: 98 (95.1%) were filled out in their entirety, and participants were able to answer the questions with a complete description of the change agent’s role and actions fresh in their minds. Because participants did not know the wide range of change agent actions prior to the training session, we believe that filling out the questionnaire at the beginning of the session would have produced results that were less representative of occupational therapists’ actual practice. However, filling it out at the end of the session may have induced a desirability bias. Nevertheless, although it relies on simple descriptive statistical analyses, this pilot study provides an initial snapshot of what occupational therapists actually do when acting as change agents and underlines the need to learn more about why they take these actions.

## Conclusion

Recent events have shown the urgency of addressing occupational injustices, which in turn points to the importance for occupational therapists of adopting a transformative approach and acting as change agents, both locally and extra-locally. Previous work on this subject mostly used a theoretical or opinion-piece lens. We believed there was a need to paint a picture of what occupational therapists actually do in their change agent practice. This pilot study shows that occupational therapists mainly act in the clinical setting, targeting their patients’ families, their own team, and their managers, using communication and collaboration actions. In this way, they act at a level that is very helpful and useful for patients and patient groups, which is commendable. Participants were less involved with non-clinical setting actors such as political stakeholders. They also used fewer mass communication actions unless they had more experience, additional education, or training. Among other hypotheses, this might suggest that with appropriate knowledge and skills, occupational therapists are willing to embrace their change agent role, whether clinical or social. As a profession, we should ensure the necessary knowledge and skills are taught, learned, and enacted. We should also consider how systemic barriers—such as NPM and neoliberal values, duty of loyalty, and lack of power—might impede full enactment of the change agent role by occupational therapists.

### Key Messages

- Providing an initial snapshot of occupational therapists’ change agent actions and the actors targeted, this pilot study shows that they mainly involve communication and collaboration in clinical settings, and target patients’ families and their own team.
- There appears to be a link between change agent actions in non-clinical settings and additional education and training.
- Further research should document what Canadian occupational therapists do in their change agent actions and what influences their choices of actions.
